# Autophagy and Lipid Metabolism as a Therapeutic Target for Overcoming Drug Resistance in Acute Myeloid Leukemia

**DOI:** 10.3390/life16030428

**Published:** 2026-03-06

**Authors:** Seyed Mohammadreza Bolandi, Mahdi Pakjoo, Briandy Fernandez-Marrero, Amir Reza Boskabadi, Erfan Mohammadi Sephavand, Jamshid Sorouri Khorashad, Saeid Ghavami, Anna M. Eiring

**Affiliations:** 1 Department of Pharmacology, Karaj Branch, Islamic Azad University, Karaj 3149968111, Iran; mreza.bolandi@gmail.com (S.M.B.); erfan.msepah94@gmail.com (E.M.S.); 2 ATMP Department, Breath Cancer Research Center, Motamed Cancer Institute, ACECR, Tehran 1517964311, Iran; pakjoomahdi@gmail.com; 3 Department of Biological Sciences, College of Science, The University of Texas at El Paso, El Paso, TX 79968, USA; bfernandez8@miners.utep.edu; 4 Faculty of Medicine, Mashhad University of Medical Sciences, Mashhad 9177948564, Iran; amirrezaboskabady@gmail.com; 5 Department of Immunology and Inflammation, Imperial College London, London W12 0NN, UK; jamshid.khorashad@icr.ac.uk; 6 Department of Human Anatomy and Cell Science, Rady Faculty of Health Sciences, Max Rady College of Medicine, University of Manitoba, Winnipeg, MB R3T 2N2, Canada; saeid.ghavami@umanitoba.ca

**Keywords:** acute myeloid leukemia (AML), autophagy, lipid metabolism, cancer drug resistance, leukemia stem cells (LSCs), non-coding RNAs

## Abstract

Acute myeloid leukemia (AML) remains a therapeutically challenging malignancy due to high relapse rates driven by leukemic stem cells (LSCs) and adaptive resistance mechanisms. Emerging evidence positions autophagy as a central regulator of AML pathobiology, exerting context-dependent effects that suppress leukemogenesis during disease initiation yet sustain LSC survival and chemoresistance in established AML. Mechanistically, autophagy integrates mitochondrial quality control, lipid droplet turnover, and metabolic rewiring to support oxidative phosphorylation, particularly under hypoxic bone marrow conditions. Lipophagy-driven fatty acid oxidation has emerged as a key metabolic vulnerability distinguishing LSCs from normal hematopoietic stem cells. Furthermore, non-coding RNAs critically modulate autophagy networks, reinforcing therapy resistance. Preclinical and clinical studies demonstrate that both inhibition and activation of autophagy may yield therapeutic benefit depending on genetic context, mutational landscape, and disease stage. We propose that integrating multi-omics approaches, particularly lipidomics, with artificial intelligence and machine learning will enable precise identification of autophagy-dependent AML subsets. Rational, biomarker-guided modulation of autophagy may overcome resistance while preserving normal hematopoiesis, offering a path toward personalized metabolic targeting in AML.

## 1. Introduction

Acute myeloid leukemia (AML) is a highly aggressive hematologic malignancy characterized by clonal expansion of immature myeloid progenitors, differentiation arrest, and poor clinical outcomes [1,2]. Standard induction therapy remains the “7 + 3” regimen, combining daunorubicin (3 days) and cytarabine (Ara-C; 7 days), followed by consolidation therapy and, when indicated, bone marrow (BM) transplantation to achieve durable remission [1,2,3,4]. Although complete remission (CR) is achievable, relapse driven by chemoresistant disease remains the principal cause of mortality. Accordingly, elucidating the molecular mechanisms underlying therapeutic resistance is central to improving AML outcomes [1,2,3,4].

Resistance in AML reflects both intrinsic leukemic programs and extrinsic BM microenvironmental influences. Signaling pathways, transcription factors (TFs), niche interactions, and non-coding RNAs (ncRNAs) have emerged as major regulatory axes. These insights have enabled the development of targeted agents, including FMS-like tyrosine kinase 3 (FLT3) inhibitors, all-trans-retinoic acid (ATRA), isocitrate dehydrogenase (IDH) inhibitors, and monoclonal antibodies [3,4]. Despite promising responses, durable efficacy remains limited, and resistance frequently develops [5,6,7,8,9,10]. Accumulating evidence identifies leukemic stem cells (LSCs) as central mediators of relapse, sustained by dynamic interactions with the BM microenvironment [4].

Autophagy has emerged as a critical adaptive mechanism within this context. As a lysosome-dependent degradative pathway, autophagy enables cellular adaptation to metabolic stress and nutrient deprivation through recycling of intracellular components [11,12]. During this process, macromolecules and organelles are sequestered within autophagosomes and delivered to lysosomes for degradation [11]. Autophagy-related gene expression is reduced in approximately 14% of AML patients [13,14]. However, paradoxically, LSCs rely heavily on autophagy as a stress-response program [1,15,16,17], with multiple studies demonstrating its association with pluripotency maintenance, self-renewal, and proliferation [18,19,20]. Under chemotherapeutic pressure, LSCs enhance autophagic capacity [17] and mitochondrial metabolism, thereby promoting survival, treatment failure, and relapse [1,15,16].

A metabolically significant extension of this process is lipophagy, the autophagic degradation of lipid droplets, which supplies free fatty acids (FFAs) to fuel fatty acid oxidation (FAO) and sustain energy production in AML cells but not in normal hematopoietic cells. This metabolic rewiring highlights a selective vulnerability within leukemic cells [21,22,23].

Importantly, autophagy exhibits context-dependent duality. Under specific therapeutic conditions, it may exert cytotoxic rather than protective effects [20]. Autophagy suppresses leukemogenesis initiation by preserving genomic stability in pre-leukemic cells [24], yet impaired autophagy may facilitate leukemic transformation, whereas excessive autophagy may promote disease progression [2,5]. These opposing roles emphasize that therapeutic targeting of autophagy must consider disease stage, genetic context, and treatment modality [2,5,25].

The pro-survival autophagic phenotype is further modulated by long non-coding RNAs (lncRNAs) [26]. LncRNAs regulate autophagy-related genes [26,27,28] and contribute to LSC-mediated drug resistance [29,30,31,32,33]. The intersection of autophagy, lipid metabolism, and lncRNA signaling therefore represents an integrated survival network that may determine leukemic cell fate [26,27].

In this review, we critically examine how autophagy, particularly lipophagy, and lncRNA-mediated regulation converge to sustain LSC survival and therapeutic resistance in AML. To provide mechanistic context, we next outline the molecular framework governing autophagy pathways.

## 2. Overview of Autophagy Pathways

**Figure 1:**
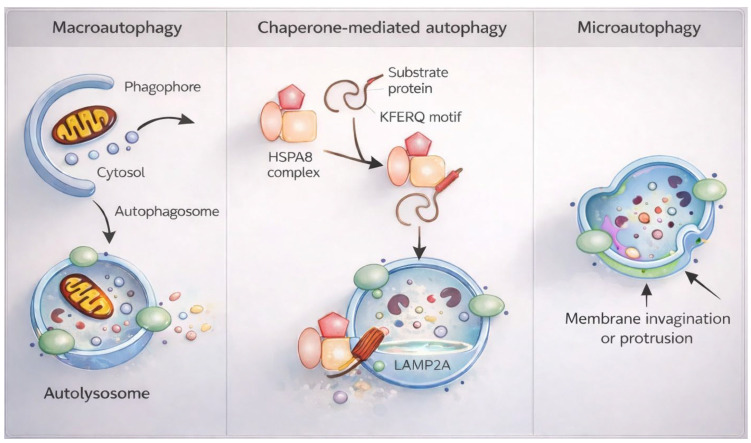
Mechanistic overview of macroautophagy, chaperone-mediated autophagy, and microautophagy. Schematic representation of the three principal autophagic pathways in mammalian cells. Macroautophagy (**left**) is initiated by formation of a phagophore that elongates to generate a double-membrane autophagosome, which sequesters cytosolic cargo and subsequently fuses with the lysosome to form the autolysosome for degradation and recycling. Chaperone-mediated autophagy (CMA) (**center**) selectively targets soluble cytosolic proteins containing a KFERQ-like motif, recognized by the HSPA8 chaperone complex and translocated across the lysosomal membrane via LAMP2A for degradation. Microautophagy (**right**) involves direct lysosomal membrane invagination or protrusion to engulf cytoplasmic components for degradation within the lysosomal lumen. Together, these pathways coordinate intracellular quality control, metabolic adaptation, and proteostasis.

The term *autophagy*, derived from the Greek words *auto* (“self”) and *phagy* (“to eat”), was first introduced by Christian de Duve in 1963. Autophagy is a lysosome-dependent degradative process that recycles intracellular components, including proteins, macromolecules, and damaged organelles, to generate metabolic precursors and sustain cellular energy homeostasis [34,35,35]. In mammalian cells, autophagy encompasses three principal forms: macroautophagy, chaperone-mediated autophagy (CMA), and microautophagy (Figure 1) [5,28,35].

**Figure 2:**
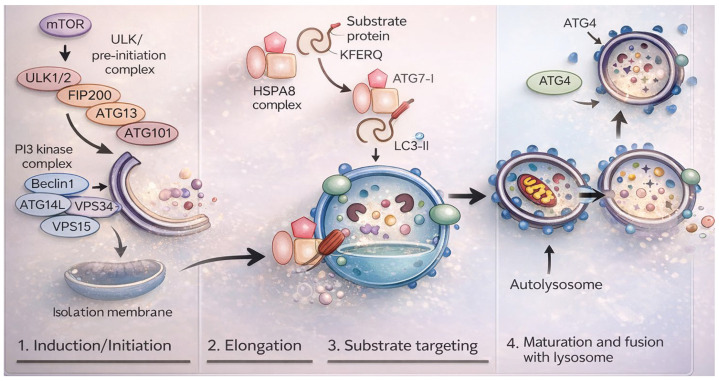
Molecular architecture and sequential stages of macroautophagy. Schematic representation of the core signaling and conjugation machinery governing autophagosome biogenesis and maturation in mammalian cells. (1) Induction/Initiation: Nutrient sensing through mTOR negatively regulates the ULK1/2 pre-initiation complex (ULK1/2–FIP200–ATG13–ATG101). Upon mTOR inhibition, the ULK complex activates the class III PI3K complex (Beclin1–VPS34–VPS15–ATG14L), promoting phosphatidylinositol-3-phosphate (PI3P) generation and phagophore nucleation. (2) Elongation: The ATG12–ATG5–ATG16L1 conjugation system and ATG7/ATG3-mediated LC3 lipidation drive membrane expansion and autophagosome formation. (3) Substrate targeting: LC3-II incorporation into the autophagosomal membrane enables selective cargo recruitment through adaptor proteins. (4) Maturation and fusion: Fully formed autophagosomes fuse with lysosomes to generate autolysosomes, where lysosomal hydrolases degrade cargo and recycle metabolites. ATG4 regulates LC3 processing during both conjugation and recycling. This coordinated cascade integrates nutrient sensing with membrane dynamics to maintain cellular homeostasis.

Macroautophagy proceeds through five coordinated stages: initiation (phagophore formation), elongation (autophagosome assembly), maturation, fusion with lysosomes, and degradation (Figure 2) [36]. The process begins with formation of an isolation membrane (phagophore), which expands into a double-membrane autophagosome that engulfs cytoplasmic cargo. Subsequent fusion with lysosomes forms the autolysosome, where degradation occurs, releasing recycled metabolites such as amino acids, nucleotides, and free fatty acids that support biosynthesis and metabolic adaptations [37].

At the molecular level, autophagosome biogenesis is orchestrated by two major protein complexes localized at the endoplasmic reticulum (ER): the Unc-51-like kinase (ULK) initiation complex and the class III phosphatidylinositol 3-kinase complex I (PI3KC3-C1). The ULK complex serves as the core initiator of autophagy and is negatively regulated by mammalian target of rapamycin complex 1 (mTORC1). Under nutrient-rich conditions, growth factor signaling through MAPK and PI3K/AKT pathways activates mTORC1 via inhibition of the TSC1/TSC2 complex, leading to phosphorylation of ULK1 and ATG13 and suppression of autophagosome formation.

During nucleation, the ULK complex recruits PI3KC3-C1 to the phagophore assembly site, where PI3KC3-C1 generates phosphatidylinositol-3-phosphate (PI3P). PI3P serves as a membrane signal that recruits PI3P-binding proteins and lipid transport machinery, including ATG2, VMP1, and TMEM41B, to drive membrane expansion. Conjugation of LC3 to phosphatidylethanolamine (forming LC3-II) enables cargo recognition through LC3–p62 interactions, thereby coupling autophagosome formation to selective substrate degradation [38,39].

Chaperone-mediated autophagy (CMA), in contrast, is highly selective and specific to mammalian cells [40]. CMA targets soluble cytosolic proteins bearing KFERQ-like motifs for lysosomal degradation via chaperone recognition and LAMP2A-mediated translocation. CMA is activated under prolonged starvation, oxidative stress, or toxic exposure [41,42,43,44]. Although CMA upregulation has been documented in several solid tumors, it appears deficient in hematologic malignancies, including AML [45].

Microautophagy represents a mechanistically distinct, generally non-selective process in which cytoplasmic components are directly internalized into lysosomes via membrane invagination or protrusion [46]. Unlike macroautophagy, microautophagy is not primarily associated with cellular stress or starvation responses [43] and involves direct uptake of cargo into late endosomes or lysosomes for degradation within the endolysosomal lumen [47].

Collectively, these autophagic pathways provide a dynamic regulatory system governing metabolic adaptation, proteostasis, and organelle quality control. In AML, the functional consequences of activating or suppressing these pathways are highly context dependent, influencing both leukemic progression and therapeutic response. This complexity necessitates a nuanced understanding of how autophagy functions within leukemic cells under treatment pressure, which is addressed in the following section.

## 3. Context-Dependent Roles of Autophagy in AML: Therapeutic Considerations

Autophagy exerts a profoundly context-dependent role in AML, functioning either as a tumor suppressor during leukemogenesis or as a pro-survival mechanism in established disease. Within the bone marrow microenvironment, characterized by metabolic stress, hypoxia, and therapeutic pressure, autophagy frequently promotes leukemic cell survival and chemoresistance by sustaining cellular homeostasis. However, in defined genetic contexts, including TP53-mutated AML, macroautophagy may exert tumor-suppressive effects [48]. These opposing functions underscore that therapeutic modulation of autophagy must be tailored to AML subtype, mutational landscape, and disease stage.

During early leukemogenesis, autophagy operates as a safeguard mechanism. In normal hematopoietic stem and progenitor cells (HSPCs), basal autophagy maintains stem cell quiescence, restricts oxidative stress, and preserves genomic integrity through mitochondrial quality control [49]. Conditional deletion or deficiency of essential autophagy-related genes, including ATG5, ATG7, and RB1CC1/FIP200, induces mitochondrial dysfunction, reactive oxygen species (ROS) accumulation, impaired myeloid differentiation, and development of myelodysplastic syndrome (MDS) with increased susceptibility to leukemic transformation [5,49,50,51]. Consistently, ATG7- or ATG5-deficient mice develop leukemia leading to premature death [17]. Autophagy further contributes to proteostatic control by degrading oncogenic fusion proteins and aggregated mutants, including FLT3 and TRAF6 mutations, the latter regulated by microRNA-146a and implicated in MDS with del(5q) or AML with normal karyotype [52]. In acute promyelocytic leukemia (APL), selective autophagy mediates degradation of the PML–RARα oncoprotein and is required for effective differentiation therapy with all-trans retinoic acid and arsenic trioxide [53,54]. Moreover, the cytotoxic effects of proteasome inhibitors such as bortezomib are dependent on autophagy induction under stress conditions in AML cells [52]. Collectively, these findings support a tumor-suppressive function of autophagy during AML initiation.

In contrast, once AML is established, autophagy is frequently co-opted to sustain leukemic persistence. Elevated autophagic flux enhances amino acid recycling, preserves mitochondrial fitness, and confers resistance to apoptosis, thereby promoting therapy resistance [5,17,19,20]. Mitophagy, the selective degradation of mitochondria, is particularly critical for leukemic stem cell (LSC) maintenance. LSCs rely on mitophagy to preserve stemness and metabolic adaptability; inhibition of mitophagy induces myeloid differentiation and compromises LSC self-renewal [19]. Autophagy activation has been associated with resistance to cytarabine, anthracyclines, and targeted therapies such as FLT3 inhibitors. Conversely, genetic or pharmacologic autophagy inhibition sensitizes AML cells to these agents in preclinical models [55]. Notably, conditional deletion of Atg5 or Atg7 increases LSC apoptosis, reduces peripheral blast counts, improves survival in leukemic mice, and enhances cytarabine efficacy, implicating autophagy in LSC-mediated chemoresistance [55].

Importantly, autophagy dependence in AML is shaped by genetic context, including FLT3-ITD and TP53 alterations, highlighting molecular heterogeneity and the necessity for stratified therapeutic approaches [56]. The hypoxic bone marrow niche further modulates autophagic activity, reinforcing stress adaptation mechanisms in both LSCs and normal hematopoietic stem cells.

**Figure 3:**
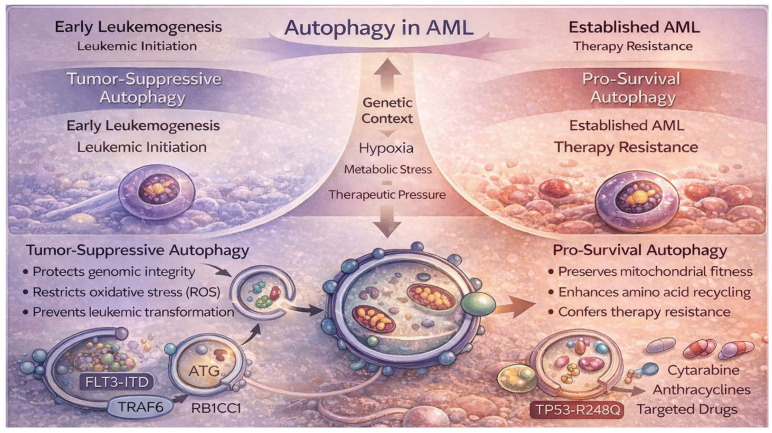
Context-dependent dual role of autophagy in acute myeloid leukemia (AML). Schematic model illustrating the bifunctional role of autophagy across AML evolution. During early leukemogenesis, basal autophagy in hematopoietic stem and progenitor cells preserves genomic integrity, limits reactive oxygen species (ROS), maintains mitochondrial quality control, and prevents malignant transformation, thereby exerting tumor-suppressive effects. Genetic disruption of core autophagy regulators (e.g., ATG5, ATG7, RB1CC1/FIP200) promotes leukemic initiation. In contrast, in established AML, particularly within the hypoxic and metabolically stressed bone marrow niche, elevated autophagic flux supports leukemic stem cell (LSC) maintenance, mitochondrial fitness, amino acid recycling, and resistance to cytotoxic and targeted therapies (e.g., cytarabine, anthracyclines, FLT3 inhibitors). Genetic context, including FLT3-ITD and TP53 alterations, modulates autophagy dependence. Together, autophagy functions as a double-edged regulator, suppressing transformation at disease onset while sustaining survival and therapy resistance in advanced AML.

Taken together, autophagy represents a double-edged regulator in AML, suppressing leukemic initiation while sustaining LSC-driven disease maintenance and therapeutic resistance. Defining the molecular determinants governing this switch is essential for rational therapeutic exploitation (Figure 3).

To dissect this survival circuitry in greater mechanistic depth, we next examine how autophagy directly drives leukemic stem cell survival and therapy resistance in AML.

### 3.1. Autophagy as a Driver of Leukemic Stem Cell Survival and Therapy Resistance in AML

LSCs exhibit high energetic demands [17] and reside within the profoundly hypoxic bone marrow (BM) niche (~1% O_2_) [25], where survival requires metabolic plasticity. Under these conditions, LSCs become critically dependent on mitophagy [57] and elevated basal autophagy [25] to sustain mitochondrial quality control and oxidative phosphorylation. Therapy-resistant AML cells exploit signaling networks, including FLT3, NF-κB [58], ATF4 [59], and PERK/NRF2 pathways [15], that converge on autophagy activation. Despite this adaptive advantage, such cells display vulnerability to lysosomal inhibition. Bafilomycin A1 (Baf A1), when combined with cytarabine (Ara-C), enhances leukemic cell death [25], supporting the rationale for combinatorial autophagy blockade in AML subsets harboring mutations such as KIT (KITD816V), STAT3, FLT3-ITD, and NPM1, all associated with increased autophagic activity [5,60,61,62].

Autophagy sustains LSC survival by buffering metabolic stress and limiting oxidative damage [5,17]. Notably, AML cells, but not normal hematopoietic counterparts, utilize autophagy-derived lipids to augment oxidative phosphorylation and overcome chemotherapy-induced stress [58]. Mitophagy-driven remodeling enhances mitochondrial efficiency and supports stemness programs [20]. Mechanistically, mitophagy upregulates mitochondrial fission 1 (FIS1), a regulator of mitochondrial dynamics and activator of glycogen synthase kinase 3 (GSK3), thereby blocking myeloid differentiation, accelerating cell-cycle progression, and reinforcing LSC self-renewal [19]. Pharmacologic disruption of mitochondrial homeostasis via autophagy inhibition demonstrates therapeutic potential in AML [25].

Genomic and transcriptional alterations further underscore autophagy dependence. Mutations in autophagy-related genes, including ATG7, RB1CC1/FIP200, and U2AF1, are reported in AML [17], while overexpression of ATG7, SIRT1, STK11/LKB1, and Beclin-1 correlates with poor prognosis and shorter remission duration [5].

Chaperone-mediated autophagy (CMA) also contributes to leukemic progression [45]. CMA selectively degrades substrates such as MLLT11/AF1q and mutant p53 via LAMP2A-mediated lysosomal targeting [63,64,65]. Moreover, PML-RARα cooperates with WDFY3/ALFY to facilitate p62-dependent autophagic degradation [66]. p62 (SQSTM1) orchestrates selective autophagy processes, including aggrephagy of oncogenic fusion proteins such as the PML-RARα [53] and pexophagy [5,67]. During pexophagy, ATM kinase recruitment to peroxisomes via PEX5 inhibits mTORC1, induces PEX5 monoubiquitination, and promotes p62-mediated autophagosome tethering [5,67]. DNA-damaging agents, including doxycycline, mitoxantrone, and etoposide, activate this pathway in AML [68]. Additionally, proteasome inhibition activates HDAC6, promoting aggresome formation and autophagic clearance of ubiquitinated proteins, thereby enabling leukemic cells to evade cytotoxic stress [69].

Mutational landscapes further modulate autophagy dependency. In NPM1-mutant AML, aberrant cytoplasmic localization of PML activates AKT signaling and PKM1/PKM2-mediated phosphorylation of Beclin-1, enhancing autophagic flux and leukemic survival [62,70]. FLT3-TKD mutations and resistance to quizartinib or sorafenib are likewise associated with elevated autophagy gene expression and increased sensitivity to autophagy inhibition [59,71]. In FLT3-ITD AML, combined VPS34 inhibition with hematopoietic mobilization (G-CSF or AMD3100) reduces leukemogenesis and LSC persistence by promoting apoptosis [72]. Increased autophagic flux has been implicated in resistance to sorafenib [73] and G9a inhibition [15]. Importantly, autophagy upregulation may reduce intracellular drug accumulation [74], whereas autophagy inhibition reverses resistance to cytarabine both in vitro and in vivo [75].

Mechanistic interplay between receptor tyrosine kinases and autophagy further influences therapeutic response. RET-mediated activation of mTORC1 inhibits FLT3 autophagic degradation, promoting survival; therefore, vandetanib or danusertib may enhance the efficacy of FLT3 inhibitors such as crenolanib [76]. Conversely, arsenic trioxide (ATO) and all-trans retinoic acid (ATRA) induce degradation of FLT3-ITD and PML-RARα via mTOR inhibition and autophagy activation, involving lncRNA HOTAIRM1 [53,77,78,79].

The KMT2A/MLL fusion protein confers adverse prognosis through ATG5-mediated autophagy [80,81,82]. In this context, autophagy inhibition may provide therapeutic benefit [5]. However, similar to PML-RARα, enhancing autophagic degradation of KMT2A fusion proteins could also represent a strategy, particularly given the frequent dysfunction of the ubiquitin–proteasome system in AML [5].

**Figure 4:**
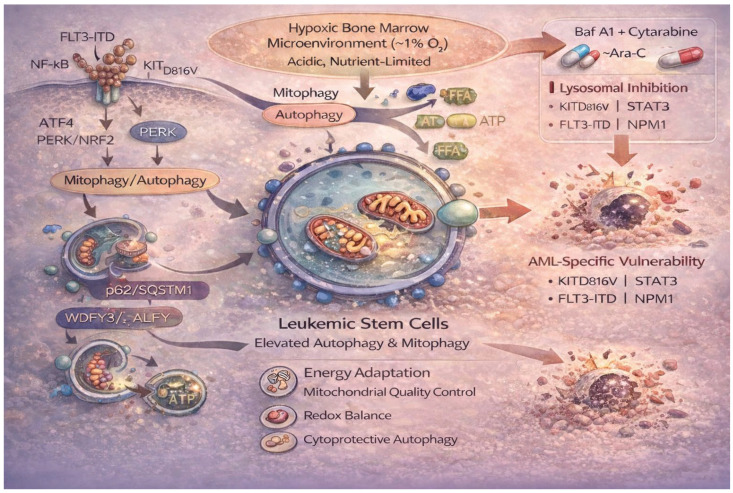
Autophagy-dependent metabolic adaptation and therapeutic vulnerability in leukemic stem cells (LSCs). LSCs exploit autophagy within the hypoxic bone marrow niche (~1% O_2_) to sustain survival and therapy resistance. Metabolic stress and oncogenic signaling pathways (FLT3, NF-κB, ATF4, PERK/NRF2) converge to activate basal autophagy and mitophagy, preserving mitochondrial quality control, redox balance, and oxidative phosphorylation. Autophagy-derived lipid recycling supports ATP production and stemness maintenance. Selective processes, including p62-mediated aggrephagy, mitophagy, and chaperone-mediated autophagy, facilitate degradation of oncogenic substrates (e.g., PML-RARα, mutant p53) and adaptation to therapeutic stress. AML subsets harboring mutations such as KITD816V, FLT3-ITD, STAT3, and NPM1 display heightened autophagic dependence. Pharmacologic lysosomal inhibition (e.g., bafilomycin A1) combined with cytarabine exploits this metabolic vulnerability, inducing leukemic cell death. The scheme highlights autophagy as a central metabolic and proteostatic axis sustaining LSC-driven chemoresistance and as a rational target for precision therapy.

Collectively, these findings position autophagy as a central metabolic and proteostatic axis sustaining LSC-driven therapy resistance. While its dual role complicates therapeutic targeting, the consistent association between heightened autophagic flux, LSC maintenance [2,5,12,16], and drug resistance provides a compelling rationale for precision-based autophagy modulation in AML (Figure 4).

Given the intimate relationship between autophagy-mediated lipid recycling and mitochondrial metabolism, we next examine the mechanistic crosstalk between autophagy and fatty acid metabolism in AML.

### 3.2. Crosstalk Between Autophagy and Fatty Acid Metabolism in AML

Intracellular lipids serve as essential energy substrates, structural membrane components, and signaling mediators, and function as precursors for bioactive molecules including hormones. Under metabolic stress, such as hypoxia or nutrient deprivation, cells mobilize free fatty acids (FFAs) to sustain ATP production, directly linking lipid metabolism to autophagic regulation. Emerging evidence demonstrates that metabolic reprogramming is a central determinant of AML progression, overall survival, and therapy resistance. Within this framework, autophagy has emerged as a critical regulator of metabolic adaptation in AML [47].

Cells metabolize stored lipids through two principal mechanisms: lipolysis and lipophagy [22,83]. In AML, autophagy and lipid metabolism are tightly interconnected, as autophagy governs lipid droplet turnover to fuel FAO and oxidative phosphorylation (OxPhos), thereby supporting leukemic cell growth and resistance to chemotherapy. This metabolic distinction between leukemic and normal hematopoietic cells provides a potential therapeutic window [84].

Fatty acids, long-chain carboxylic acids, constitute fundamental energy substrates and membrane building blocks [85]. While normal hematopoietic stem cells (HSCs) predominantly rely on glycolysis for energy production [86], LSCs display a functional dependency on FAO [84]. In contrast to HSCs, LSCs rely heavily on mitochondrial OxPhos and are unable to compensate via glycolysis when mitochondrial respiration is impaired [87]. Notably, in de novo AML, OxPhos in LSCs is primarily fueled by amino acids; however, at relapse, metabolic reliance shifts toward FAO, contributing to reduced sensitivity to agents such as venetoclax and azacitidine [88,89]. This metabolic plasticity underscores the adaptive capacity of LSCs under therapeutic pressure.

Beyond energy production, fatty acids influence membrane fluidity, oxidative stress responses, and integrated stress signaling networks [90]. Lipid droplets function as dynamic organelles maintaining lipid homeostasis by storing neutral lipids for subsequent mobilization. They buffer oxidative stress by limiting lipid peroxidation and provide substrates for membrane biosynthesis and signaling molecules [91]. Lipophagy, a selective autophagic process, facilitates the sequestration of lipid droplets into autophagosomes and their lysosomal degradation into FFAs via lysosomal lipases [92,93]. Increasingly recognized as a pivotal regulator of cellular metabolic fitness [21], lipophagy supplies fatty acids for β-oxidation and mitochondrial ATP production.

**Figure 5:**
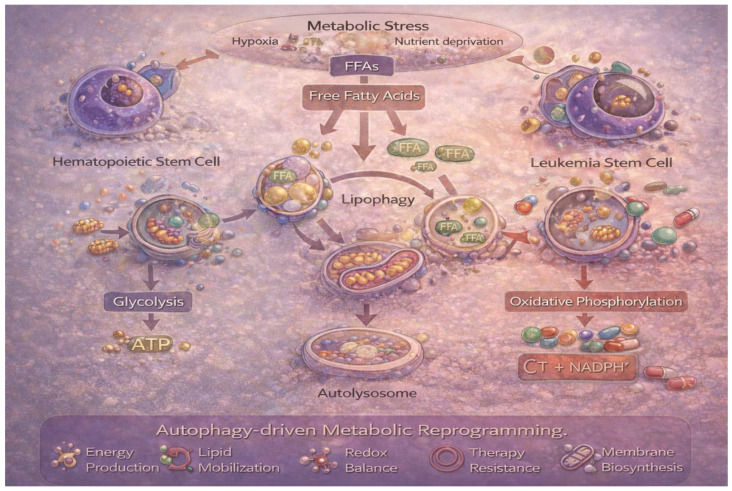
Autophagy–lipid metabolic coupling drives oxidative phosphorylation and therapy resistance in AML. Metabolic interplay between autophagy and fatty acid metabolism in acute myeloid leukemia (AML). Under hypoxia and nutrient deprivation within the bone marrow niche, lipid droplets are mobilized through lipophagy to generate free fatty acids (FFAs), which fuel β-oxidation and mitochondrial oxidative phosphorylation (OxPhos). While normal hematopoietic stem cells primarily rely on glycolysis, leukemia stem cells (LSCs) exhibit FAO-dependent OxPhos, particularly at relapse, promoting survival and resistance to agents such as venetoclax and azacitidine. Autophagy-mediated lipid recycling supports ATP production, redox balance, membrane biosynthesis, and stress adaptation. Inhibition of autophagy disrupts this metabolic axis, leading to lipid accumulation, impaired mitochondrial respiration, and increased therapeutic vulnerability. This model highlights lipophagy-driven metabolic reprogramming as a selective vulnerability in AML.

In AML, lipid-fueled OxPhos is critical for proliferation, survival, and stress tolerance, particularly during chemotherapy. Autophagy inhibition disrupts this metabolic axis, leading to lipid accumulation, impaired OxPhos, and heightened vulnerability in mitochondria-dependent leukemic cells [24,47,94]. Consequently, lipid droplets and lipophagy have emerged as promising metabolic vulnerabilities linked to chemosensitivity and treatment response (Figure 5).

Given the tight coupling between autophagy-driven metabolic rewiring and therapeutic resistance, regulatory layers controlling this axis warrant deeper investigation. Among these, non-coding RNAs have emerged as pivotal modulators of autophagy and metabolic adaptation in AML.

### 3.3. Non-Coding RNAs, Autophagy, and Drug Resistance in AML

Drug resistance in AML arises through multiple coordinated mechanisms, including enhanced drug efflux, accelerated cell-cycle progression, repair of damaged organelles, evasion of apoptosis, and alterations in drug targets and metabolism [95]. Non-coding RNAs (ncRNAs) participate in each of these processes. Although only 5–10% of the human genome encodes proteins, the majority is transcribed into ncRNAs [96,97,98], which are broadly characterized by size and function.

Small ncRNAs (sncRNAs), shorter than 200 nucleotides, include microRNAs (miRNAs), piwi-interacting RNAs (piRNAs), and tRNA-derived stress-induced RNAs (tiRNAs). Long ncRNAs (lncRNAs), exceeding 200 nucleotides, encompass large intergenic non-coding RNAs (lincRNAs) and transcribed ultra-conserved regions (T-UCRs) [99]. Functionally, ncRNAs are classified as housekeeping (e.g., tRNA, rRNA, snRNA) or regulatory (e.g., miRNAs, circRNAs, siRNAs, piRNAs, and lncRNAs) [99,100]. These molecules regulate transcription, RNA processing, translation, post-translational modification, and epigenetic remodeling [101,102]. Aberrant ncRNA expression contributes to AML drug resistance through dysregulation of oncogenes, tumor suppressors, transcription factors, signaling pathways, and relapse following remission [103].

LSCs, which sustain self-renewal and therapy resistance, represent a principal barrier to durable remission [104,105]. ncRNAs critically regulate autophagy in LSCs [106,107], thereby influencing metabolic adaptation and chemoresistance. miRNAs modulate autophagy by targeting core autophagy proteins such as Beclin-1 or signaling pathways including AMPK–mTOR [107,108]. For example, miR-30a suppresses autophagy via Beclin-1 downregulation, whereas miR-138 activates the AMPK–mTOR axis [109,110].

Among lncRNAs, DANCR promotes cytarabine (Ara-C) resistance in AML by sponging miR-20a-5p and activating the miR-874-3p/ATG16L1 axis, thereby enhancing cytoprotective autophagy [111]. Similarly, downregulation of miR-143 correlates with cytarabine resistance through reduced autophagy inhibition [112]. HOTAIRM1 functions as a key autophagy regulator [26], mediating ATRA-induced differentiation and PML–RARα degradation in acute promyelocytic leukemia (APL) via autophagy activation. Mechanistically, HOTAIRM1 sponges miR-20a/106b and miR-125b, upregulating E2F1, DRAM2, and ULK1 [79]. HOTAIRM1 knockdown enhances Ara-C cytotoxicity by modulating Wnt/β-catenin signaling, an established regulator of autophagy [79,113].

HOTAIRM1 is also linked to adriamycin resistance in AML and other malignancies [104,105,114,115]. Through AKT/Notch1 activation and p21 suppression, it promotes proliferation and multidrug resistance [116]. Notably, Notch signaling mediates stromal–leukemic interactions that enhance survival [117], highlighting the importance of microenvironmental crosstalk.

The BM microenvironment further shapes ncRNA–autophagy interactions. Stromal cells suppress miR-23a-5p via NF-κB activation, leading to TLR2 upregulation and protective autophagy induction in leukemic cells. Elevated miR-23a-5p enhances anthracycline sensitivity and improves responses to daunorubicin and arsenic trioxide (ATO) when combined with autophagy inhibitors such as hydroxychloroquine (HCQ) or bafilomycin A1 [7]. DANCR similarly enhances LSC quiescence and self-renewal during cytarabine therapy through miR-20a-5p and miR-874-3p suppression and ATG16L1 upregulation [111].

Additional ncRNAs promote autophagy-mediated resistance. lncRNA AK156230 activates ULK2, ATG7, and ATG16L; NBR2 directly activates AMPK; Ad5-AlncRNA and PTENP1 suppress PI3K/AKT/mTOR; and HOTAIRM1, PTENP1, and MALAT1 upregulate ULK [118]. Conversely, lncRNA Risa, MEG3, H19, and miR-30a-5p inhibit autophagy by suppressing MTDH and Akt signaling [33,118]. miR-17HG, negatively regulated by miR-21 and reduced in AML, enhances apoptosis via PTEN overexpression, thereby restoring chemosensitivity [119].

Collectively, ncRNAs integrate autophagy signaling with metabolic adaptation, microenvironmental cues, and survival pathways in AML. This regulatory layer provides a mechanistic bridge between stemness, metabolic rewiring, and therapy resistance, positioning ncRNA–autophagy networks as promising therapeutic targets. Given this mechanistic foundation, strategic modulation of autophagy emerges as a rational therapeutic approach in AML.

## 4. Autophagy Modulation as a Therapeutic Strategy

### 4.1. Preclinical Evidence for Autophagy-Targeted Therapies

Accumulating preclinical evidence establishes autophagy as a critical determinant of AML progression and therapeutic response [5]. Pharmacologic modulation of autophagy, either inhibition or activation, has emerged as a promising strategy to enhance treatment efficacy. However, clinical translation remains complex due to toxicity concerns, compensatory resistance mechanisms, patient heterogeneity, and the essential role of autophagy in hematopoietic stem cell (HSC) repopulation following remission [5]. Inappropriate suppression of autophagy may impair normal HSPC recovery, while insufficient autophagic flux can paradoxically enhance tumorigenesis [17].

A broad spectrum of autophagy-modulating agents has been developed, targeting lysosomal acidification (H^+^ pumps), HSP70/90, cathepsins, mTORC1/2, p140, and PI3K pathways [120]. Chloroquine (CQ) and hydroxychloroquine (HCQ), the only clinically approved autophagy inhibitors, block lysosomal acidification and autophagosome–lysosome fusion [121], thereby inhibiting late-stage autophagy. These agents have been evaluated in AML for their antitumor activity [122]. However, CQ/HCQ exhibit autophagy-independent effects, including TNF-α downregulation [123] and NOTCH1 activation that may stabilize the tumor microenvironment [124]. Their requirement for high dosing [17,121] and TP53-dependent efficacy [125] further limit clinical potency, particularly in TP53-mutated AML. Consequently, more potent agents such as Lys05 [126], the VPS34 inhibitor PIK-III, and ROC-325, which synergizes with azacitidine, are under investigation [127].

Autophagy inhibition enhances the activity of epigenetic therapies, including bromodomain and extraterminal (BET) inhibitors [128]. While mature AML blasts are sensitive to JQ1, LSCs exhibit resistance. In LSCs, JQ1 induces protective autophagy characterized by increased Beclin-1 expression, LC3-II lipidation, autophagosome formation, and reduced p62 levels [128]. Concurrent activation of AMPK (pThr172) and ULK1 (pSer555) implicates the AMPK/ULK1 axis as a therapeutic vulnerability [128], particularly given AMPK’s role in maintaining low ROS and sustaining LSC self-renewal [19].

HDAC inhibitors (HDACis), including valproic acid (VPA), vorinostat (SAHA), trichostatin A (TSA), panobinostat, and givinostat promote autophagy in leukemia [5], including AML1-ETO-rearranged leukemia where autophagy promotes survival [129]. Combination strategies pairing HDACis with autophagy inhibitors may overcome resistance [129]. Conversely, in pediatric AML-M7, low basal autophagy renders HDACi treatment cytotoxic through ROS accumulation [130,131]. Notably, vorinostat efficacy can be attenuated by therapy-induced autophagy [132].

Similarly, the AKT inhibitor perifosine demonstrates therapeutic promise in AML but induces compensatory autophagy-mediated resistance [132]. Co-treatment with 3-methyladenine (3-MA) restores cytotoxicity, reinforcing the rationale for combinatorial inhibition [62].

Conversely, selective autophagy induction may promote degradation of oncogenic drivers, including mutant TP53, KMT2A fusions, FLT3-ITD, and PML::RARA [125]. For example, TP53^R248Q accumulation enhances autophagy, and HSP90 inhibition (17-AAG) may facilitate its degradation via autophagy or CMA [65]. Wild-type TP53 activation under genotoxic stress increases autophagy-dependent cell death through DRAM1, SESN1/2, and ULK1 upregulation [5,133]. Targeting casein kinase 1α (CK1α), which interacts with MDM2 and regulates AMPK/mTOR signaling, represents another strategy to induce cytotoxic autophagy (Table 1).

Collectively, preclinical data reveal that autophagy modulation is highly context-dependent: in some AML subsets, autophagy functions as a cytoprotective mechanism requiring inhibition; in others, therapeutic benefit may derive from enforced autophagic degradation of oncogenic proteins. These mechanistic insights from preclinical models provide a rationale for clinical evaluation of autophagy regulators, either as monotherapy or in combination regimens.

### 4.2. Clinical Trials Highlighting the Effects of Autophagy Regulators in AML Treatment

Multiple clinical trials have evaluated autophagy-modulating agents in AML (Table 1), reflecting increasing recognition of autophagy as a therapeutic target. However, outcomes have been heterogeneous, underscoring the importance of molecular stratification and disease context.

Statins, classically used to reduce plasma cholesterol through inhibition of hydroxy-3-methylglutaryl-CoA reductase (HMG-CoAR), also exert anti-leukemic effects by modulating proliferation, migration, and apoptosis via miRNA-dependent pathways. Simvastatin promotes miR-19a-3p–mediated degradation of HIF-1α [152] and preclinical studies demonstrate synergy with mTOR and aminopeptidase inhibitors [153,154]. Clinically, the addition of pravastatin to cytarabine and idarubicin achieved acceptable response rates in a phase II study [155]. However, subsequent evaluation failed to meet predefined efficacy thresholds, and further development was not recommended [156]. These mixed results highlight the complexity of repurposing metabolic agents in AML.

mTORC inhibitors, including sirolimus, everolimus, and temsirolimus, have also been tested clinically. Sirolimus combined with the MEC regimen (mitoxantrone, etoposide, cytarabine) improved response rates in high-risk non-M3 AML without increasing toxicity [157]. In contrast, everolimus added to low-dose cytarabine showed no survival benefit in elderly AML patients [158] and post-induction everolimus failed to improve outcomes in a randomized study [159]. These findings suggest that mTOR inhibition may benefit selected high-risk subgroups but lacks universal efficacy.

Bortezomib, a proteasome inhibitor, induces apoptosis partly through modulation of autophagy in AML cells [160,161,162,163]. In relapsed/refractory (R/R) AML, combination therapy with bortezomib, homoharringtonine, and cytarabine demonstrated tolerability and higher complete remission rates in FLT3-mutant patients [164]. However, in older AML patients without FLT3 mutations, adding bortezomib to decitabine did not improve remission or survival [165], potentially reflecting low proteasome subunit expression [166]. Additional studies yielded inconsistent benefits [167] whereas incorporation of bortezomib into MEC achieved a 56.5% complete remission rate in R/R AML [168]. Collectively, these data indicate that bortezomib efficacy may depend on mutational context and proteostatic dependency.

Histone deacetylase inhibitors (HDACis), including vorinostat, trichostatin A, chidamide, and panobinostat, induce apoptosis and autophagy in AML cells [141]. Panobinostat combined with cytarabine/idarubicin achieved a 64% complete response rate and median overall survival of 17 months in elderly patients [169], with manageable toxicity. A separate phase I trial demonstrated improved survival when panobinostat was added to induction therapy [170]. Chidamide combined with decitabine increased remission rates but not overall survival [171]. Vorinostat combinations demonstrated activity but were limited by toxicity [54], and dosing schedule significantly influenced tolerability [172]. These findings reinforce the need to balance autophagy modulation with toxicity management.

Venetoclax, a BCL-2 inhibitor, directly induces apoptosis and Beclin-1–dependent autophagy by disrupting the BCL-2/Beclin-1 complex [145]. Multiple studies confirm its efficacy in AML. A meta-analysis demonstrated superior remission rates for venetoclax plus azacitidine compared to azacitidine alone, albeit with increased adverse events [173]. Venetoclax combined with hypomethylating agents showed improved remission and event-free survival compared to cytarabine-based regimens [174]. Addition of venetoclax to standard 7 + 3 induction significantly improved remission rates and overall survival [175], and venetoclax-based intensive regimens facilitate transition to allogeneic HSC transplantation. Meta-analyses further support venetoclax combined with low-dose cytarabine or hypomethylating agents for patients unfit for intensive therapy [176]. Given its dual capacity to trigger apoptosis and modulate autophagy, BCL-2 inhibition represents one of the most clinically validated autophagy-linked strategies in AML.

Overall, clinical experience confirms that autophagy modulation influences therapeutic response, but efficacy is highly dependent on genetic context, disease stage, and combination strategy. Rational integration of autophagy-targeted approaches therefore requires biomarker-driven patient selection and mechanistic precision. These clinical insights set the stage for a forward-looking discussion on optimizing autophagy modulation in AML.

## 5. Conclusions and Future Directions

Autophagy represents a central adaptive axis in AML, functioning as both a tumor suppressor during leukemogenesis and a metabolic survival mechanism in established disease. Its intimate integration with leukemic stem cell (LSC) maintenance, mitochondrial fitness, and lipid-driven oxidative phosphorylation underscores its role in therapy resistance. Overcoming resistance therefore requires precision modulation rather than indiscriminate inhibition.

Future strategies should integrate multi-omics profiling, including transcriptomics, proteomics, metabolomics, and particularly lipidomics, to define autophagy dependence at the patient level. Lipidomic signatures can identify reliance on lipophagy-driven fatty acid oxidation (FAO), enabling stratification of AML subsets vulnerable to metabolic disruption. Given the metabolic shift toward FAO at relapse, longitudinal lipidomic monitoring may predict therapeutic escape and guide adaptive interventions.

Artificial intelligence (AI) and machine learning (ML) offer transformative potential in this context. Integrative ML models can combine genomic mutations (e.g., FLT3-ITD, TP53), autophagy-related gene expression, lipidomic profiles, and clinical response data to identify predictive biomarkers of autophagy addiction. Deep-learning approaches may further uncover nonlinear interactions between lipid metabolism and autophagic flux that are not apparent through conventional analyses.

Therapeutically, rational combination strategies, pairing autophagy inhibitors or inducers with targeted agents, epigenetic drugs, or BCL-2 inhibitors, should be guided by systems-level modeling rather than empirical design. Ultimately, overcoming AML resistance will require dynamic, data-driven therapeutic algorithms that leverage OMICS-informed stratification and AI-guided personalization to selectively disrupt autophagy-dependent survival networks while preserving normal hematopoiesis.

## Figures and Tables

**Table 1:** The effect of different therapeutics on autophagy in AML cells.

Family	Drug	Autophagy Status	Mechanism	Ref
mTORC Inhibitors	Rapamycin Temsirolimus	Increase in protective autophagy	Induce autophagy by blocking phosphorylation of the inhibitory site serine 757 on the kinase ULK1 (a key autophagy regulator) in AML cells	[17,134,135]
Statins	Simvastatin Lovastatin Atorvastatin Rosuvastatin	Increase in protective autophagy	Induce autophagy by downregulating Akt/mTOR/p70S6K signaling.	[136]
Recombinant Arginase	HuArgI(Co)-PEG5000	Increase in protective autophagy	mTORC1 inhibition	[137,138]
NOTCH Inhibitors	(Small-molecule γ-secretase inhibitors) GSIs	Increase in protective autophagy	mTORC1 inhibition	[139,140]
Proteasome Inhibitors	Bortezomib	Increase in protective autophagy	mTORC1 inhibition	[11]
Histone Deacetylase Inhibitors	Trichostatin A Vorinostat Entinostat Butyric Acid	Increase in autophagy (protective/cytotoxic)	mTORC1 inhibition and FOXO1 activation	[141,142]
BET ^1^ Inhibitors	JQ1	Increase in protective autophagy	mTORC1 inhibition and AMPK/ULK1 activation	[128,143]
Chemotherapy Agents	Doxorubicin Mitoxantrone Etoposide Daunorubicin Cytarabine	Increase in protective autophagy	Increased p62 and decreased Bcl-2	[5,68,144]
RET	-	Increase in cytotoxic autophagy	Aggrephagy of mutated proteins	[76]
ATO ^2^	-	Increase in cytotoxic autophagy	Aggrephagy of mutated proteins	[77]
G9a ^3^ Inhibitors	-	Increase in protective autophagy	PERK/NRF2 signaling protects LSCs against ROS-induced apoptosis	[5,15]
BCL2 Inhibitors	Venetoclax	Increase in protective autophagy	Activate Beclin-1-dependent autophagy	[5,145,146]
Rapamycin Analogs	Sirolimusm Temsirolimus Everolimus	Increase in cytotoxic autophagy	Sensitizing AML subtypes to ATRA	[5,147]
Calcium Channel Blockers	Verapamil Loperamide Pimozide	Increase in cytotoxic autophagy	Sensitizing AML subtypes to ATRA	[5,148,149]
Lithium	-	Increase in cytotoxic autophagy	Reduces autophagy in cancer cells, sensitizes AML subtypes to ATRA, and promotes myeloid differentiation of AML cells	[5,150]
Multi-BCR:ABL1 and SRC Family Tyrosine Kinase Inhibitors	Dasatinib	Increase in cytotoxic autophagy	Promoting myeloid differentiation of AML cells	[5,151]

^1^ BET: Bromodomain and extraterminal domain; ^2^ ATO: Arsenic Trioxide; ^3^ G9a: Histone Methyltransferase.

## Data Availability

Not applicable.
